# Coelenterazine Analogs for Bioassays and Molecular Imaging

**DOI:** 10.3390/s25061651

**Published:** 2025-03-07

**Authors:** Sung-Bae Kim, Genta Kamiya, Tadaomi Furuta, Shojiro A. Maki

**Affiliations:** 1 Environmental Management Research Institute (EMRI), National Institute of Advanced Industrial Science and Technology (AIST), 16-1 Onogawa, Tsukuba 305-8569, Japan; 2 Department of Engineering Science, Graduate School of Informatics and Engineering, The University of Electro-Communications, Chofu 182-8585, Japan; kamiya0801@uec.ac.jp (G.K.); s-maki@uec.ac.jp (S.A.M.); 3 School of Life Science and Technology, Institute of Science Tokyo, B-62 4259 Nagatsuta-cho, Midori-ku, Yokohama 226-8501, Japan; furuta@bio.titech.ac.jp

**Keywords:** bioluminescence, coelenterazine, imaging, bioassay, luciferase

## Abstract

Coelenterazine (CTZ) is a common substrate of marine luciferases upon emission of bioluminescence (BL) in living organisms. Because CTZ works as a “luminophore” in the process of BL emission, the chemical modification has been centered for improving the optical properties of BL. In this review, we showcase recent advances in CTZ designs with unique functionalities. We first elucidate the light-emitting mechanisms of CTZ, and then focus on how the rational modification of CTZ analogs developed in recent years are connected to the development of unique functionalities even without luciferases, which include color tunability covering the visible region, specificity to various proteins (e.g., luciferase, albumin, and virus protein), and activatability to ions or reactive oxygen species (ROS) and anticancer drugs. This review provides new insights into the broad utilities of CTZ analogs with designed functionalities in bioassays and molecular imaging.

## 1. Introduction

A myriad number of light-emitting species exist in nature and are especially common in the oceans. This bioluminescence (BL) from living organisms is a cold light-emitting event caused by the oxidation of a small molecular weight substrate called luciferin, catalyzed by an enzyme named luciferase inside living organisms [1].

As the key components for BL, both luciferases and luciferins are equally important, but recent studies have focused on the genetic engineering of luciferases rather than the chemical modification of luciferins. This genetic engineering has contributed to the creation of new mutants with excellent optical properties or the development of molecular imaging probes, such as bioluminescence resonance energy transfer (BRET) [2,3] and protein-fragment complementation assay (PCA) probes [4,5]. In parallel, the authors note the benefits of chemical modification of luciferins, although they have attracted relatively less attention to date.

Luciferin is an essential component for BL as the energy source and is supplied by the biosynthesis in living organisms. However, most biosynthesis mechanisms are unknown, although those of fungal luciferin [6] and bacterial luciferin [7] have been partly elucidated. In contrast to the variety of light-emitting species in nature, their luciferin repertoires are extremely small, because few luciferins are shared by many luciferases. The representative ones include (i) beetle luciferin (d-luciferin) for beetle luciferases, (ii) coelenterazine (CTZ) for marine luciferases, (iii) tetrapyrrole luciferin, (iv) fungal luciferin, (v) bacterial luciferin, and (vi) *Cypridina* luciferin [8].

In this review, the authors focus on CTZ and its analogs developed in recent years, and showcase the light-emitting mechanisms, recent advances in the chemical designs, and the corresponding versatility in bioassays and molecular imaging.

## 2. Coelenterazine as a Common Substrate of Marine Luciferases

Luciferin is a key component for developing BL with luciferases. The role of luciferins has been explained as “bioluminophore”, because it determines the colors and works as the energy source through its oxidization catalyzed by luciferases.

The luciferin in the aqua phase is diffused into the active site of the specific luciferase to work as a “bioluminophore”, whereas the “fluorophore” of fluorescent proteins is derived from an intrinsic part of the FP. The most common luciferin of marine luciferases, CTZ, chemically resembles the fluorophore of fluorescent proteins (most often a derivative of the tripeptide sequence ^65^XYG^67^), sharing the same imidazolone ring structure [9]. Because of its uniqueness as a potential optical platform, CTZ has been centered on organic modification by appending unique functional groups to the backbone of CTZ.

This section mainly focuses on the chemical basis of CTZ and its recent studies on the analogs that are connected to the applications to bioassays and molecular imaging.

### 2.1. Chemical Structure of Coelenterazine

In the early 1960s, Dr. Johnson’s group at Princeton University isolated the Ca^2+^-sensitive photoprotein aequorin from jellyfish [10]. Later, they isolated two compounds, now called coelenteramine (CTM) and coelenteramide (CTMD), from aequorin [11]. CTZ was originally isolated as an actual substance from the liver of the luminous squid *Watasenia scintillans*, and was chemically synthesized by Inoue et al. for the first time [12]. The occurrence of CTZ is extremely widespread among marine bioluminescent organisms. However, CTZ has not been found in terrestrial creatures [1].

Marine luciferins such as *Cypridina* luciferin (Vargulin) and CTZ are biosynthesized in living organisms, and their chemical structures are derived from amino acids, that is, actually modified tripeptides that are made from arginine, isoleucine, and tryptophan (RIW) in the case of *Cypridina* luciferin [13,14]; and one phenylalanine and two tyrosine residues (FYY) in the case of CTZ [1,15], respectively.

### 2.2. Chemiluminescence-Emitting Mechanism of CTZ

**Figure 1:**
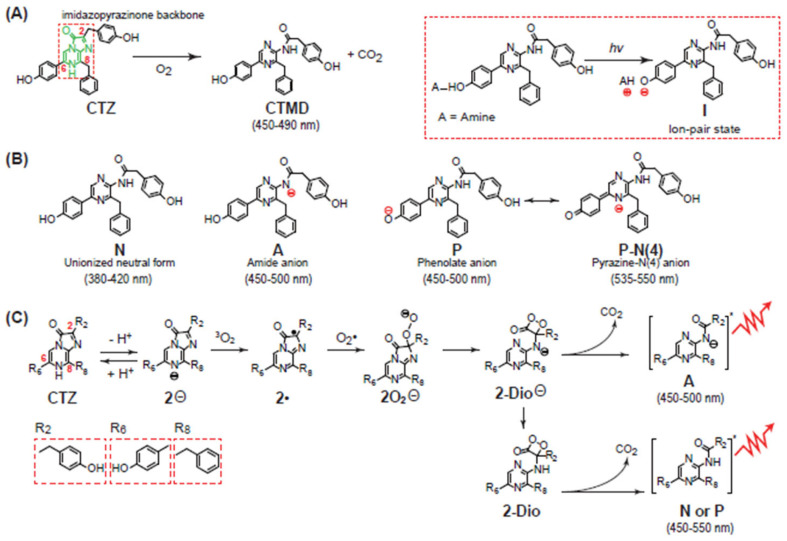
(**A**) The imidazopyrazinone structure of CTZ and its oxidative reaction to generate luminescence, giving coelenteramide (CTMD). (**B**) The intermediate forms of CTZ in the process of the oxidative reaction with molecular oxygen. (**C**) The detailed mechanism of chemiluminescence emission of CTZ through its oxidative reaction.

CTZ produces luminescence in the process of an oxidative decarboxylation reaction of its imidazopyrazinone structure. Briefly, CTZ is peroxidized by molecular oxygen (O_2_). The peroxide forms an amide anion of coelenteramide (CTMD) in its excited state, resulting in the emission of blue luminescence (typically λ_max_ 450~490 nm) (**Figure 1A**) [1]. In the process, it is known that CTZ forms five potential intermediate light emitters: unionized neutral form (**N**), phenolate anion (**P**), ion-pair state (**I**), amide anion (**A**), and pyrazine-N(4) anion (**P-N(4)**).

Among the intermediates, the most common light emitter is the amide anion (**A**). Other light emitters such as phenolate anion (**P**) and its resonance structure called pyrazine-N(4) anion (**P-N(4)**) can be generated according to the experimental conditions and luciferases. The neutral form (**N**) light emitter can be made in the case that the OH group at the C-6 position is replaced with other functional groups. This neutral form (**N**) emits the shortest wavelength luminescence (generally λ_max_ 380–420 nm). The phenolate anion (**P**) and amide anion (**A**) generate slightly longer wavelength luminescence than that of the neutral form (**N**), typically λ_max_ 450–500 nm. The longest wavelength luminescence is created by the pyrazine-N(4) anion (**P-N(4)**), generally λ_max_ 535–550 nm [16]. The phenolate anion temporarily formed from the ion-pair state (**I**) can be made only by light excitation; thus, it is very exceptional and can be excluded from the list of the emitter forms. The phenolate anion (**P**) and its resonance structure pyrazine-N(4) anion (**P-N(4)**) are practically the same intermediate (**Figure 1B**).

The first rational studies on the light-emitting mechanism of marine organisms began with *Cypridina* luciferin [17], which was the first marine luciferin isolated and shares the same imidazopyrazinone backbone as that of CTZ.

*Cypridina* luciferin can emit light in two different ways: chemiluminescence (CL) induced by aprotic solvents; and BL catalyzed by luciferases, whose mechanisms are known to be similar to each other [18]. The mechanism of the CL emission is explained below.

As illustrated in **Figure 1C**, the light-emitting reaction of CTZ in aprotic solvent starts via deprotonation of the N-7 nitrogen to form a CTZ anion (**2^⊖^**) that reacts directly with O_2_ at the C-2 carbon to form a CTZ peroxide ion (**2O_2_^⊖^**). Following the oxygen addition, the resulting nucleophilic peroxide moiety performs an intramolecular attack on the C-3 carbonyl to form a cyclic dioxetanone intermediate (**2-Dio^⊖^**). Subsequent ring decomposition via decarboxylation leads to the loss of CO_2_ and the formation of an anionic excited-state aminopyrazine product (coelenteramide ion) in a singlet excited state (**A**). This unstable anionic species then decomposes with the emission of light, resulting in CL. Alternatively, the cyclic dioxetanone intermediate (**2-Dio^⊖^**) can be protonated (**2-Dio**) and form the neutral (**N**) or phenolate anion intermediates (**P**) after losing CO_2_. These unstable anionic species then decompose with the emission of light [19].

### 2.3. Bioluminescence-Emitting Mechanism of Coelenterazine

This section reviews the light-emitting mechanism of BL. Recently, Schenkmayerova et al. presented a unique approach for investigating the light-emitting mechanism of CTZ using a unique non-oxidative CTZ analog called azaCTZ and X-ray crystallographic information of *Renilla* luciferase 8 (RLuc8) and its ancestor enzyme AncFT [20]. The mechanism basically follows the same process as that of the CL as described above.

**Figure 2:**
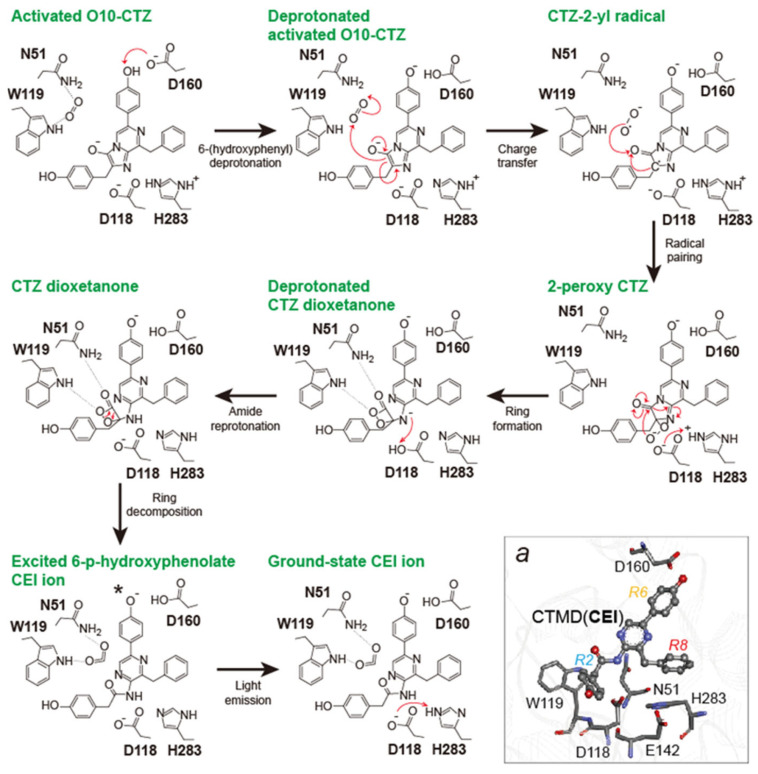
A proposed catalytic mechanism of CTZ-powered *Renilla*-type bioluminescence. This figure was modified from a reference [19]. Inset *a* shows the binding mode between *Renilla*-type luciferase (AncFT) and CTMD (CEI) based on the PDB structure, 7QXQ. This tertiary structure shows involvement of D160 besides the catalytic pentad, N51, D118, W119, E142, and H283.

The proposed BL-emitting mechanism is as follows (**Figure 2**): CTZ enters the enzyme active site with a deprotonated imidazopyrazinone core because the pKa of the core (7.55) is very close to the physiological pH. Upon binding, the -OH group of the C-6-(p-hydroxyphenyl) substituent is deprotonated by D160 to form the activated dianion **O10-CTZ**, which affects the emission maximum (λ_max_). In the ternary Michaelis complex, the side chains of N51 and W119 position a co-substrate molecule (dioxygen) such that it can be directly attacked by the C-2 carbon of **O10-CTZ**. Their initial interaction occurs via a charge-transfer radical mechanism. The next step involves radical pairing and termination to form the **2-peroxy-CTZ** anion, which then cyclizes via a nucleophilic addition-elimination mechanism. This step yields a highly unstable and energetic dioxetanone structure of CTZ with a deprotonated amide group. At this point, the amide group is protonated by D118 to avoid significant attenuation of the BL signal due to the formation of the deprotonated **CTMD** product. D118 also prevents hydrolysis of the amide bond by a water molecule, which yields the side-product coelenteramine (CTM). After reprotonation, the unstable dioxetanone ring decomposes and the released energy excites the newly formed **CTMD (CEI)** ion. As it returns to the ground state, the excited molecule releases a photon, representing the BL signal, together with the final products, ground-state **CTMD** ion and carbon dioxide (CO_2_). The residue H283 is reprotonated via an interaction with E142.

## 3. Creation of Coelenterazine Analogs

Although CTZ is the common substrate for marine luciferases, it practically has intrinsic limitations in its application to bioassays and molecular imaging.

For example, (i) CTZ has relatively poor solubility and is chemically unstable in physiological samples [21]; (ii) the optical intensity of CTZ is still not strong enough for challenging molecular imaging missions including animal imaging; (iii) the color variation in CTZ is limited to blue and green that is easily attenuated by physiological samples and tissues [22]; and (iv) the conventional role of native CTZ is limited to luminesce with marine luciferases, and does not address the necessities of additional functionalities such as analyte sensing and conditional signal reporting.

**Figure 3:**
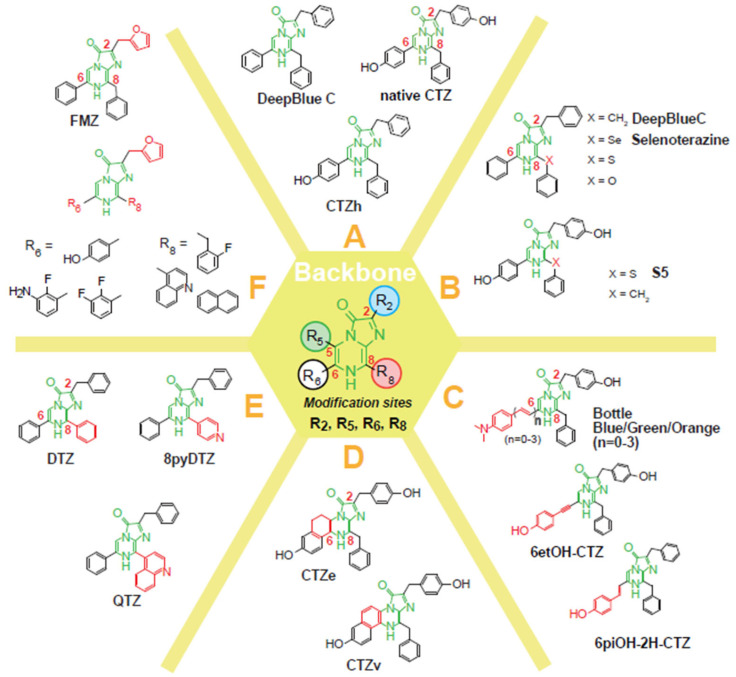
Overview of the CTZ analogs with typical modification at the imidazopyrazinone backbone. The C-2, C-5, C-6, and C-8 positions of the backbone were modified for better optical properties. Group A represents the historically old and structurally simple chemical CTZ analogs; Group B shows CTZ analogs with a specific elbow at the C-8 position; Group C categorizes CTZ analogs with a π-electron extension at the C-6 position; Group D represents CTZ analogs with a bridge connecting the C-5 and C-6 positions; and Groups E and F are designed for FMZ analogs.

To date, CTZ has been chemically modified to employ additional optical properties or functionalities, besides the conventional role. Upon synthesis of CTZ analogs, the imidazopyrazinone backbone of CTZ itself has not been modified because it serves as the scaffold of the oxidation reaction. Instead, the functional groups at the C-2, C-5, C-6, and C-8 positions of the backbone have been chemically engineered as a way to improve the optical properties [23]. The CTZ analogs were briefly categorized into six groups according to the chemical structural similarity as illustrated in **Figure 3** (named Groups A–F).

### 3.1. Categorization of Coelenterazine Analogs

Among many CTZ analogs, Group A represents historically old and structurally simple chemicals (Group A in **Figure 3**): (i) Coelenterazine h (CTZh) was originally reported by Hori et al. in 1973 [24], where the absence of the OH group at the C-2 position is the different feature from CTZ. It is known that CTZh generates strong BL intensities with NanoLuc, RLuc derivatives, and ALuc. CTZh does not show a significant luciferase specificity, but is the most widely used among CTZ analogs; and (ii) DeepBlueC (DBC) is a well-known CTZ analog that is characteristic in the absence of the OH group at both the C-2 and C-6 positions, compared to CTZ [25]. It is known that DBC luminesces in blue with NanoLuc and RLuc. The far blue-shifted peak advantageously works for transferring the resonance energy to fluorescent proteins in BRET systems.

**Figure 3** briefly illustrates the overall modification of the C-2, C-6, and C-8 positions of the imidazopyrazinone backbone of CTZ: Group B represents the elbow-modified CTZ analogs at the C-8 position. Group C shows the π-electron conjugation prolonged CTZ analogs at the C-6 position. Group D is characteristic of CTZ analogs with a bridged form at the C-5 and C-6 positions. Groups E and F were commonly designed for NanoLuc and its derivatives, where Group E represents CTZ analogs with a benzyl group at the C-2 position and phenyl group or pyridyl group at the C-6 position, whereas Group F is unique in having a furfuryl group at the C-2 position.

It is known that the designs of Groups B–F contribute to red-shifted BL and those of Groups C–F are effective for luciferase specificity. The designs of Groups E–F are focused on enhancing both BL intensities and spectral redshifts of NanoLuc. The distinctively designed CTZ analogs are surprisingly tolerated by marine luciferases. Each case is explained in detail in the following subsections.

### 3.2. Color-Tunable Coelenterazine Analogs

CTZ and its analogs generate unique colors according to which of the five intermediates are predominately made during the oxidative reaction catalyzed by marine luciferases as explained above. For example, a red-shifted BL is expected to be with the pyrazine-N(4) anion (**P-N(4)**) intermediate, whereas a blue-shifted BL is with the neutral form (**N**) intermediate.

Technically speaking, a significantly blue-shifted BL can be made by modifying the OH group at the C-6 position of CTZ, because this modification creates the neutral form (**N**) intermediate during the reaction with RLuc derivatives [26].

**Figure 4:**
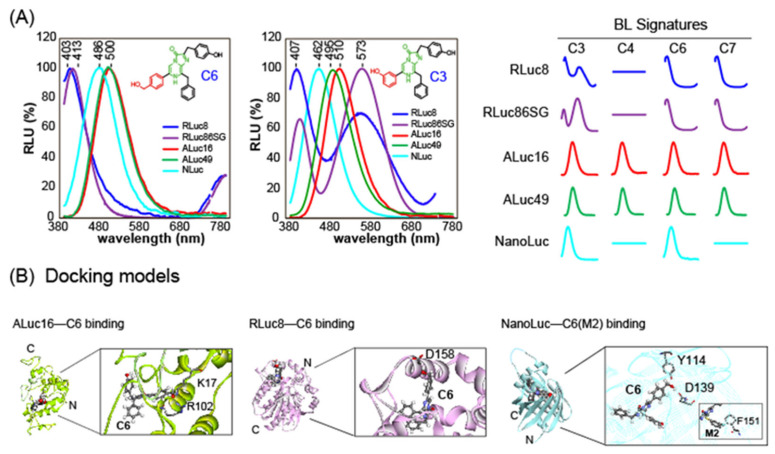
(**A**) BL spectra of CTZ analogs, **C3** and **C6**, according to marine luciferases. The right panel showcases the spectral signatures of representative C-series CTZ analogs that represent each marine luciferase. (**B**) Binding of selected CTZ analogs with marine luciferases, explaining the spectral signatures.

This mechanism of blue-shifted BL spectrum fits well with RLuc derivatives, but not necessarily with the other marine luciferases. For example, a C-6 position-modified CTZ analog named **C6** (R6 = Bnz-*p*-CH_2_OH) shows a significantly blue-shifted BL spectrum especially with RLuc8 and RLuc86SG (λ_max_ ca. 403–413 nm), whereas the same CTZ analog generates moderate blue (λ_max_ ca. 465 nm) and greenish blue (λ_max_ ca. 500 nm) BL spectra with the other marine luciferases NanoLuc and ALuc16, respectively [27]. This result indicates that the intermediates of the CTZ analog **C6** are differently formed according to marine luciferases (**Figure 4A**).

Another CTZ analog **C3** (R6 = Bnz-*m*-OH) shows much more diverse shapes of BL spectra according to marine luciferases. The CTZ analog interestingly develops two separated peaks (blue and orange) in the BL spectrum with RLuc8 and RLuc86SG, although it generates single-peaked BL spectra (bluish green) with the other marine luciferases. This feature indicates that **C3** generated two major intermediates during the reaction with RLuc derivatives, whose ratios are various according to RLuc derivatives. Because of the uniqueness in the BL spectral shapes of **C3**, one can easily distinguish which luciferase is applied to BL imaging. Kamiya et al. previously described the “spectral signature” for identifying each reporter luciferase and these substrates were named C-series CTZ analogs [27].

This spectral uniqueness of CTZ analogs is also supported by the distinctive binding modes of the substrate **C6** with marine luciferases, suggesting that the oxidative reaction environments forming the intermediates inside luciferases should be distinctive from each other (**Figure 4B**).

**Figure 5:**
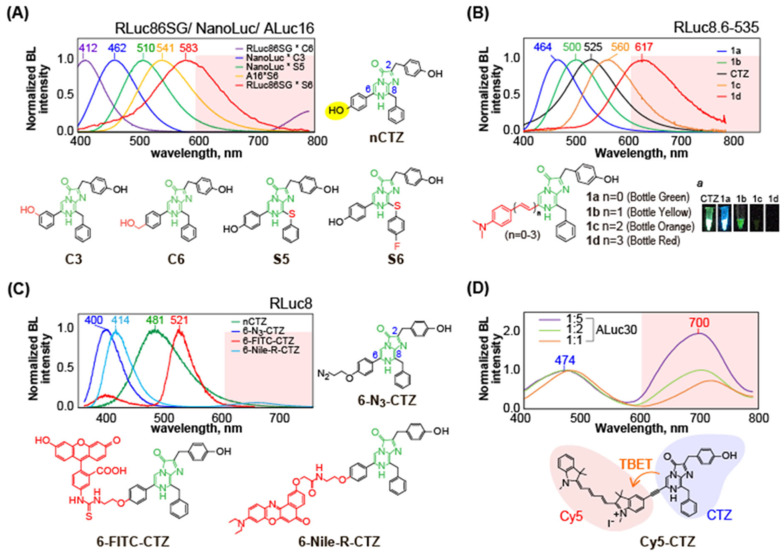
(**A**) BL spectra of C- and S-series CTZ analogs according to marine luciferases. (**B**) Diverse BL spectra of bottle-series CTZ analogs with RLuc8.6-535. (**C**) Dye-conjugated CTZ analogs. The red colored frames represent the fluorescent dyes. (**D**) The relative TBET spectra of Cy5-CTZ with ALuc30, where the peak heights were normalized to the peak height contributed by the CTZ part (474 nm) in the green region. The legend indicates the relative ratios of the luciferase (left) to the substrate (right). The numbers on the peaks denote the maximal wavelengths (λ_max_) of the spectra.

BL colors covering the visible region can be intentionally created in a way to combine specific CTZ analogs with marine luciferases. **Figure 5A** shows the typical combinations of the CTZ analog and marine luciferase, where S-series CTZ analogs emit relatively red-shifted BL spectra compared to C-series CTZ analogs. This occurs because the carbon ankle of the benzyl group at the C-8 position is replaced with sulfur (S) for extending the π-electron conjugation [28].

The most common and straightforward way to redshift a BL spectrum is to extend the π-electron conjugation of the imidazopyrazinone backbone (Group C in **Figure 3**). Based on this idea, Tamaki et al. introduced a series of CTZ analogs in a way to increase the number of double bonds for extending the π-electron conjugation at the C-6 position of the imidazopyrazinone backbone [29]. This approach was commonly effective for exerting the red shifts in the BL spectra of any marine luciferases (**Figure 5B**).

Furimazine (FMZ) is characteristic with respect to the absence of the OH group at the C-6 position and the substitution of the phenol group with a furan group at the C-2 position upon comparison with CTZ (Group F in **Figure 3**) [30]. FMZ is specific to NanoLuc and has proved its excellent optical intensities in various molecular imaging modalities to date. However, FMZ is known to be toxic in cells and animal models and is poorly soluble, thus, limiting the doses in physiological systems [31]. To address the limitations, various FMZ analogs have been reported to date, e.g., fluorofurimazine (FFz) that is brighter than FMZ with improved solubility [32] and cephalofurimazine (CFz) for bright imaging of molecular events in the brain [33].

Extending π-electron conjugation at the C-8 position of the backbone of FMZ is also known to be effective for red shifts in BL spectra especially with NanoLuc and its derivatives. For example, Shakhmin et al. reported a series of FMZ analogs. Among them, installation of a 4-quinoline group to the C-8 position of FMZ analogs exerted red-shift emission by ca. 100 nm while maintaining reasonable light output [34]. Similarly, pyridyl and 4-quinoline groups can be introduced to the C-8 position of a FMZ analog, diphenylterazine (DTZ), to create 8pyDTZ and QTZ, respectively [35].

Another effective method to create red-shifted BL with CTZ analogs is conjugating fluorescent dyes to the C-2 or C-6 position of the backbone of native CTZ (Group C in **Figure 3**) [36]. Nishihara et al. synthesized a series of dye-conjugated CTZ analogs: ones conjugated by Fluorescein isothiocyanate (FITC) (6-FITC-CTZ), Nile red (6-Nile-R-CTZ), and Cyanine-5 (Cy5) at the C-6 position (Cy5-CTZ). As mentioned above, C-6 position-modified CTZ analogs generally show blue-shifted BL spectral peaks at around 400 nm with RLuc derivatives. A C-6-modified analog, 6-FITC-CTZ, showed a unique spectrum peak at 522 nm with RLuc8.6-535, in addition to that at 400 nm. Among the synthesized dye-conjugated CTZ analogs, 6-Nile-R-CTZ showed a characteristic resonance energy transfer peak at 650 nm, where ca. 11% of the total photons were emitted in the red region for longer than 600 nm, which is highly tissue-permeable and commonly referred to as the “optical window” (**Figure 5C**) [37].

In addition, Cy5-CTZ was developed by attaching Cy5 dye to CTZ through an acetylene linker. This linker enables through-bond energy transfer (TBET) between the energy donor CTZ and the energy acceptor Cy5. This Cy5-fused CTZ analog is intrinsically fluorescent and emits near-infrared (NIR)-shifted luminescence through Cy5 upon reacting with appropriate luciferases, RLuc and ALuc (**Figure 5D**) [38]. The authors demonstrated that Cy5-CTZ is optically stable in physiological samples, easily passes through the plasma membrane, and emits NIR BL in animal cells.

### 3.3. Luciferase-Specific Coelenterazine Analogs

Although BLs have diverse colors ranging from blue to red according to luciferin–luciferase combinations, most of the peak emissions populate the blue-yellow region [39,40]. Furthermore, the spectra have broad bandwidths and, thus, overlap with each other. This feature causes optical cross-leakage that severely impairs multiplex imaging. To tackle this issue, BL systems with two or more multiple luciferase reporters conventionally make use of quenchers, optical filters, and/or deconvolution schemes so as to discriminate the multiple BL signals [41,42]. However, this cannot be considered as the fundamental solution to the issue.

In order to address the intrinsic drawback of BL, some researchers redesigned CTZ analogs so that they have luciferase specificities in multi-reporter assay systems.

**Figure 6:**
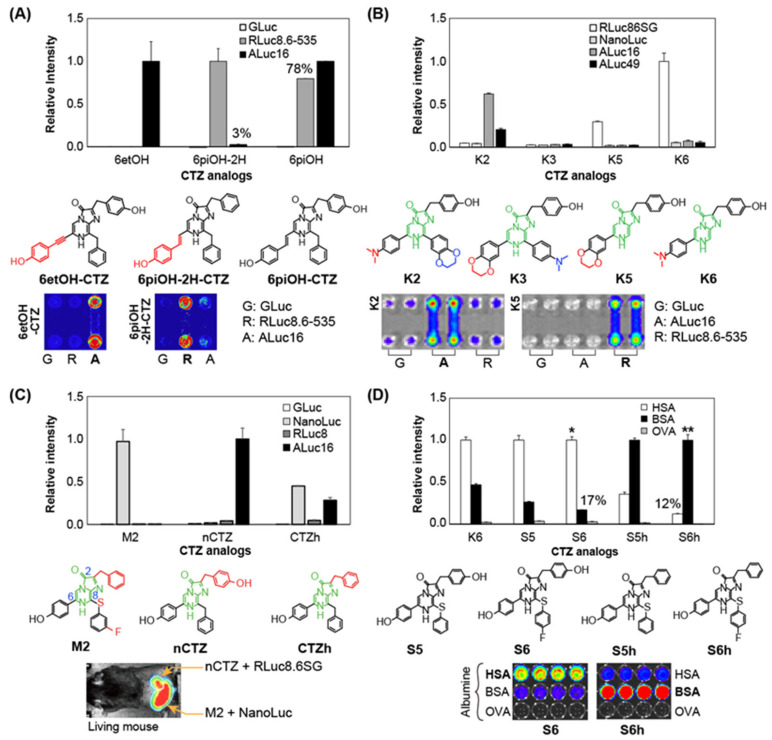
(**A**) Relative luciferase specificity of representative CTZ analogs, 6et-OH-CTZ, 6pi-OH-2H-CTZ, and 6pi-OH-CTZ. The bottom panel shows the corresponding chemical structures of the substrates. The imidazopyrazinone backbone was highlighted in green and the characteristic functional groups were marked in red. (**B**) Relative luciferase specificity of K-series CTZ analogs according to marine luciferases. The overall BL intensities were depicted in relative values compared to that of the substrate **K6** with RLuc86SG. (**C**) Comparison of the luciferase specificity of **M2** with those of nCTZ and CTZh. (**D**) Relative luminescence intensities of S-series CTZ analogs in response to various serum albumins. Abbreviations: HSA, human serum albumin; BSA, bovine serum albumin; OVA, ovalbumin.

Nishihara et al. previously synthesized 20 kinds of CTZ analogs with modifications to the C-2 and C-6 functional groups (Group C in **Figure 3 and Figure 6A**), some of which developed luciferase-specific BL signals [43].

For example, ethynylation at the C-6 position of the imidazopyrazinone backbone (i.e., 6etOH-CTZ) is specific only to a marine luciferase ALuc16, whereas an introduction of a double bond (olefin) at the C-6 position (e.g., 6piOH-CTZ) exerts its preferable binding to both RLuc8.6-535 and ALucs. It is considered that the double bond addition and orientation at the C-6 position determines the structural preference for marine luciferases in addition to the red shifts in BL. CTZ analogs without the OH group at the C-2 position (e.g., 6piOH-2H-CTZ) specifically, luminesced only with a marine luciferase RLuc8.6-535, but ones having the OH group at the C-2 position (e.g., 6piOH-CTZ) did not. This specificity indicates that the OH group at the C-2 position is a key functional group in binding to ALucs; however, it is not essential for interaction with RLuc8.6-535. It is explained that the C-2 position is located at the loose moiety of RLuc8.6-535 and, thus, is less influential, compared to the case of ALuc [43].

Kamiya et al. also reported a new series of luciferase-specific CTZ analogs (named **K1**–**K6**) with modification of the C-6 and C-8 positions [44] (**Figure 6B**): (i) by modification of the π-electron conjugation through replacement of the benzyl group with a phenyl group at the C-8 position of the imidazopyrazinone backbone and (ii) by introduction of a dimethylaminophenyl group or a bulky benzodioxane group to the C-6 or C-8 positions of the backbone. The functional group-deficient CTZ analogs at the C-8 position, named **K5** and **K6**, are effective for the RLuc specificity, whereas a CTZ analog carrying a bulky group at the C-8 position, named **K2**, is specific to ALucs.

In parallel, Kamiya et al. reported a series of regiospecific CTZ analogs, where all the functional groups at the C-2, C-6, and C-8 positions of the imidazopyrazinone backbone were regiochemically modified, and found that some of the analogs are highly specific to NanoLuc. For example, **M2** is a regiospecific CTZ analog carrying a *m*-fluorobenzenethiol group at the C-8 position and without the OH group at the C-2 position, and specific to NanoLuc [45] (**Figure 6C**). The authors’ computational modeling explained the NanoLuc specificity of **M2** with the following reasons: (i) the benzyl group at the C-2 position is positioned at the hydrophobic region of NanoLuc and (ii) the *m*-fluorobenzyl group at the C-8 position of **M2** is in a suitable position to form a CF–π interaction with the benzyl group of F151 of NanoLuc (**Figure 4B**, right inset).

Some CTZ analogs with unique functional groups can be specific to serum albumins from different animal species (**Figure 6D**). Based on this concept, Nishihara et al. reported a CTZ analog for imaging human serum albumin (HSA) [46]. They reported only an HSA-specific CTZ analog emitting luminescence in blue. Independently, Kim et al. reported such unique CTZ analogs that specifically luminesce with bovine and mouse serum albumins (BSA and MSA) as well as HSA as pseudo-luciferases emitting red-shifted BL [47]. The kinetic constants of serum albumins as pseudo-luciferases are reported in references using conventional methods of enzyme kinetics. However, the specific mechanism of the substrate oxidation and light emission has not yet been elucidated. In the CTZ analog design, Kim et al. paid attention that the functional group at the C-8 position of CTZ was less investigated. They finally found that the C-8 position of CTZ is the key for the specificity with albumins and red shifts in the spectra. In their design on the CTZ analogs, the original carbon elbow of the benzyl group at the C-8 position was replaced with sulfur (S) for extending the *π*-electron conjugation. In addition, the *para*-position of the benzyl structure at the C-8 position was optionally modified with fluorine (F). It was found that the CTZ indicators immediately luminesce bluish green and yellowish green with HSA and BSA, respectively. This study shows that CTZ analogs work as simple and less-invasive optical indicators for serum albumins, which can recognize specific albumins and report the red-shifted luminescence signals.

In parallel, it was also reported that *Cypridina* luciferin, sharing the same imidazopyrazinone backbone, is reactive with the spike proteins of COVID-19 (SARS-CoV-2) acting as a pseudo-luciferase [48].

### 3.4. Activatable Coelenterazine Analogs

**Figure 7:**
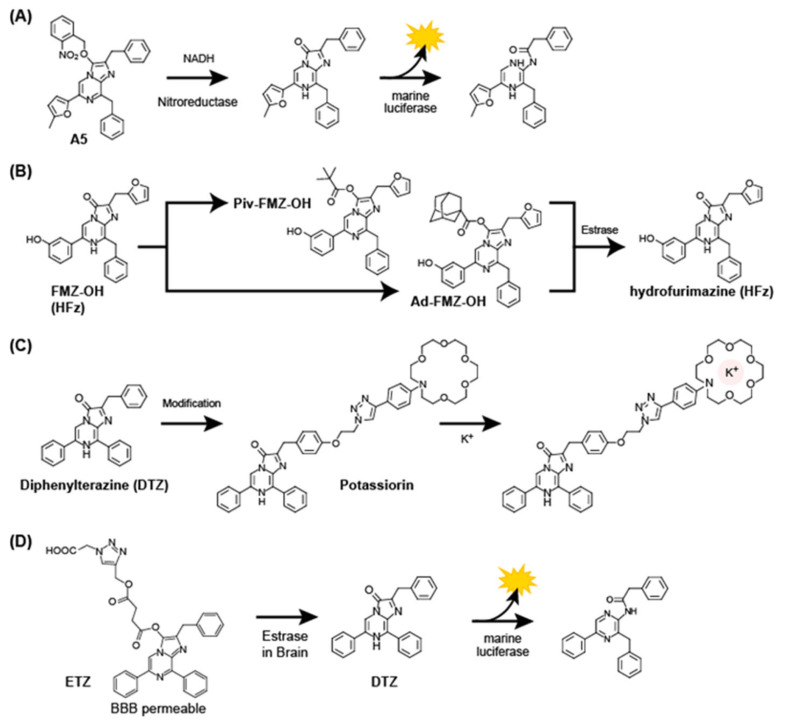
(**A**) BL-emitting mechanism of a caged CTZ analog **A5** for determining nitroreductase activity. (**B**) BL-emitting mechanism of a caged CTZ analog, Piv-FMZ-OH and Ad-FMZ-OH, derived from FMZ-OH (Hydrofurimazine; HFz) for determining esterase activity. (**C**) Design of an analog named Potassiorin that is derived from the diphenylterazine (DTZ) structure. The C-2 position of DTZ is modified with a K^+^-binding crown ether ring to derive Potassiorin. (**D**) Modification of the C-3 position of the DTZ backbone for improving the BBB permeability and minimizing BBB efflux.

The CTZ analogs described above passively work as substrates of conventional luciferases or pseudo-luciferases like albumins. However, some researchers studied potential active use of CTZ analogs that have additional abilities such as responding to a specific stimulator (ligand or specific wavelength light) or recognizing a target protein and quantitatively reporting optical signal(s), in addition to their conventional role as luciferase substrates. Prototypically activatable CTZ analogs are the ones that embed a conceptional “on-off switch”. For this purpose, a chemical modification of the functional groups of the imidazopyrazinone backbone of CTZ with a cage was attempted (**Figure 7**).

Caged luciferins are typical activatable means, where the substrate itself is protected by a bulky cage and released by a specific signal or molecular event [49]. These caged luciferins commonly carry a bulky group, which blocks the interaction of the core imidazopyrazinone backbone with luciferases. The cages were designed to be removed by specific wavelengths of UV irradiation in animal models [50], a stimulator (analyte) including oxygen species [51], copper ions [49,52], calcium ion [53], and labile iron levels [54]. Similarly, a caged CTZ analog was reported to quantitatively image nitroreductase (NTR) activity, where the C-3 position of the imidazopyrazinone structure of the CTZ analog was modified with a nitrobenzyl moiety. Because NTR reduces the functional group with the nitrobenzyl moiety to the aniline group, net CTZ analog is released and catalyzed by marine luciferase to emit BL [55].

FMZ has also been targeted for the caging of the functional groups, because it carries the common imidazopyrazinon backbone. A series of caged FMZ analogs were developed by introducing a protective group at the C-3 position and an OH group at the C-6 position of the backbone. The protective groups are cleaved by esterase inside living animal cells to generate the deprotected forms, which work as the substrates of the specific luciferase, NanoLuc [56]. Among the series, the analogs with the bulkiest protecting group (adamantanecarbonyl group) and a hydroxy substituent (named Ad-FMZ-OH) showed significantly prolonged and constant BL signal in animal cells expressing NanoLuc, compared to the native FMZ substrate.

Protection of the C-3 position of a FMZ analog DTZ with a carboxylate functional group (named ETZ) is known to enhance both water solubility and the blood–brain barrier (BBB) permeability, and reduce BBB efflux [53]. When ETZ is delivered in the brain, nonspecific esterase hydrolyzes the linker ester bind of the protective group, releasing free DTZ for BL reaction.

Recently, a unique ion-selective CTZ analog named “Potassiorin” was developed for imaging potassium ion (K^+^) levels in mammalian cells [57]. The CTZ analog was made by replacing the OH group at the C-2 position with a K^+^-binding moiety known as a “crown ether” structure. This analog significantly reduces the BL intensity after K^+^ binding.

These activatable CTZ analogs have unique advantages compared to conventional passive ones: One can expect minimized autoluminescence with such activatable CTZ analogs. This is because the embedded on–off switch has the effect of reducing the background signals in basal conditions. For example, caged luciferin remains protected until an enzymatic cleavage event occurs and, thus, it minimizes autoluminescence.

### 3.5. Coelenterazine Analogs as Reactive Oxygen Species Scavenger and Anticancer Drugs

**Figure 8:**
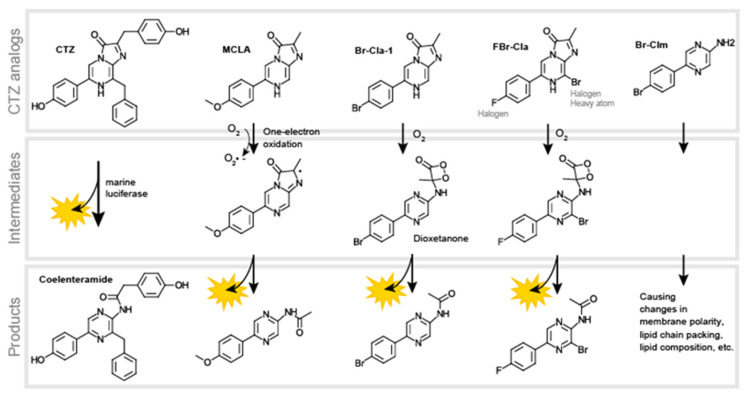
Chemical structures of CTZ analogs working as ROS scavengers and anticancer drugs. Native CTZ (left end in the upper panel) is modified to generate ROS scavengers (MCLA) and cancer drugs (Br-Cla-1 and FBr-Cla). The corresponding reactions produce oxidated compounds in the bottom panel.

Aerobic organisms oxidize energy substrates with O_2_ and generate ATP. In the process, reactive oxygen species (ROS), superoxide radical anion, or hydrogen peroxide are inevitably generated. It is known that the physiological levels of ROS can be quantitatively imaged with CTZ analogs such as *Cypridina* luciferin analog (CLA) and Cypridina luciferin methoxy analog (MCLA). The working mechanism basically follows an oxidation process of the CTZ analogs for producing CL [58], i.e., MCLA reacts with ROS leading to the formation of the intermediate in the excited state. The intermediate relaxes to the ground state with the emission of CL (**Figure 8**).

Recently, CTZ analogs have been focused on anticancer activities. For example, brominated CTZ analogs were developed for self-activating photodynamic therapy of cancer (PDT) [59,60]. The CTZ analogs were further modified not to be activated by light irradiation but by superoxide anion [61], which is overproduced in cancer cells.

The anticancer effect of brominated CTZ analogs is explained as the bromine heteroatoms increase chemiexcitation to triplet states, not singlet states, due to the heavy-atom effect [62]. The chemiexcited triplet CTZ analogs generate highly cytotoxic singlet oxygen species to kill cancer cells after reaction with O_2_. Moreover, a brominated CTZ analog named Br-Clz is specifically cytotoxic to cancer cell lines such as prostate, breast, lung, gastric, and neuroblastoma cell lines, but not to normal breast cell lines [59]. The CTZ analog was further modified by fluorine at the C-2 position (named FBr-Cla) and found to maintain anticancer activity [63]. The same authors also developed a brominated analog of coelenteramine named Br-Clm, which has an aminopyrazine core instead of an imidazopyrazinon one but possesses higher anticancer activity toward gastric and lung cancer lines [64]. The working mechanism of Br-Clm was not elucidated, but the same author suggested that the anticancer effect of Br-Clm is due to significant changes in cellular membrane lipids in gastric cancer cells, which could be attributed to its anticancer mode of action [65]. Because CTZ is derived from tripeptides, it is basically nontoxic in living subjects. However, the above CTZ analogs are exceptionally toxic to a cancer cell line through specifically generating cytotoxic singlet oxygen species in the process of their chemiexcitation [66] or causing significant changes in cellular membrane lipids.

As exemplified above, expanding the role of CTZ analogs to anticancer drugs shows the versatility of CTZ analogs carrying an imidazopyrazinone structure. The examples show that CTZ analogs can provide a breakthrough in cancer and cell-signaling research, considering that ROS commonly causes pathogenic cellular damage events and physiological cellular redox signaling and regulation [58].

## 4. Conclusions

The recent innovative research on BL has greatly diversified the repertories of luciferases and luciferins in the toolbox. While both luciferases and luciferins are equally important as the key components for BL, the authors have observed that the major portion of recent studies have been focused on luciferases rather than luciferins and the versatility of luciferins have been underestimated, compared to that of luciferases.

This review showcased the diverse utilities of the common marine luciferin, CTZ, and its analogs, which range from the roles of conventional substrates of luciferases to activatable BL indicators, ROS scavenger, and even anticancer drugs. Considering the versatility of CTZ and its analogs to date, future studies should be devoted to expanding the utilities from developing the basic materials to advancing the functionalities. Future studies may include expanding the color palettes and developing NIR BL systems. The direct merits of these developments could be that one can construct advanced bioassay systems that can determine ligands or molecular events that have not been assayed before, while the use of red and NIR BL can enhance imaging applications in physiological samples and in living subjects.

In light of these efforts, BL should be defined by being truly quantitative, highly sensitive, and working as versatile optical readouts. Such breakthroughs in BL will be achieved from ideas inspired by the understanding of nature, artificial intelligence (AI)-assisted computer modeling, and even bold imagination.

## Data Availability

No new data were created in this review article.
